# The Texas Medical College

**Published:** 1893-02

**Authors:** 


### THE TEXAS MEDICAL COLLEGE.

It will be remembered that the JOURNAL has pointed out the fact that the comparatively large fees charged for tuition in the Medical Department of the University of Texas (Galveston), was operating to prevent even Texas students from matriculating there, and recommended that, at least until the school should have secured a firm footing, it would be best to reduce the fees very materially. The JOURNAL, while not advocating, by any means, free medical education, pointed out that in all other departments of the Texas University there was no charge, or a nominal charge, for tuition, insisted on a reduction of the fee to at least that charged by many other Southern Colleges—$50 per session.

It is therefore with much pleasure we learn that the Board of Regents, seeing that the second annual class was no larger than the first, appreciated the fact that the charge for tuition was detrimental to the best interests of the school, and have accordingly, —not reduced it,—but abolished it altogether; and now a payment of $30 on matriculating, will entitle the student to attend the three years courses, there being no other charge, we believe, except the usual fee for diploma, the last year. This, of course, no student who has successfully gone through the three courses and the green room, will grumble at paying.

The change, of course, does not apply to the present session; those who have matriculated and paid the first year's tuition are not entitled to get any of it refunded, as we understand; the change will take effect next September; but those who have paid this session will have nothing to pay the next and the next, as we understand it.

Thus, the main objection having been removed; the Col[illegible] being now fairly under way, with elaborately equipped laboratories and a large corps of eminent, experienced and able teachers, paid by the State and in no way dependent upon revenue derived from students, we look to see the institution flourish and prosper.

Texas furnishes a very large contingent of medical students annually, and there is now no longer any necessity or excuse for a single one of them to go beyond the State line for a medical education of the highest order.

